# Implementation of remote units in two large out-of-hours emergency primary care districts in Norway

**DOI:** 10.1080/02813432.2025.2470470

**Published:** 2025-02-28

**Authors:** Erik Zakariassen, Steinar Hunskaar

**Affiliations:** a National Centre for Emergency Primary Health Care, NORCE, Norwegian Research Centre, Bergen, Norway; b Group for Health Services Research, Department of Global Public Health and Primary Care, University of Bergen, Bergen, Norway; c Centre for Prehospital and Emergency Research and Danish Center for Health Services Research, Aalborg University Hospital and Department of Clinical Medicine, Aalborg University, Aalborg, Denmark

**Keywords:** Out-of-hours care, casualty clinic, prehospital emergency care, telemedicine, Norway

## Abstract

**Objective:**

An inter-municipal out-of-hours (OOH) district covers several municipalities with one centralized casualty clinic. These OOH districts are large geographical areas with long driving times to the casualty clinic. Remote OOH units were established in two OOH districts in Norway, to secure better access to the OOH service. Patients were offered video consultations with nurse-led appointments at the remote OOH units. The aim was to investigate contact rates and distribution of consultation types before and after the remote units were established. Design. An observational study with pre- and post-data collected from municipalities with and without (controls) remote OOH units.

**Setting:**

Two OOH districts, Førde and Molde, with five and four remote OOH units, respectively.

**Subjects:**

Inhabitants contacting the Local Emergency Communications Centers (LEMCs) in the two areas.

**Results:**

In municipalities that established remote OOH units the contact rates to the LEMCs decreased by 15% in Førde and 16% in the Molde OOH districts in 2021, compared with 2019. Control municipalities had an increased rate of 7% and 2%, respectively. Consultation rates decreased by 16% and 12% in municipalities with remote OOH units in Førde and Molde, respectively. In 2021, 7% of contacts from municipalities with remote OOH units in Førde OOH district and 3% in Molde OOH district ended in a consultation at a remote OOH unit. In the Molde OOH district, where the traditional casualty clinic was replaced with remote OOH units, home visits and callouts decreased by 76% and 86% from 2019 to 2021.

**Conclusion:**

Establishing remote OOH units could have decreased contact and consultation rates in both districts. Most contacts were handled with actions other than a remote OOH unit encounter with video consultation. There was a large reduction of home visits and callouts in the Molde OOH district in 2021, compared with 2019.

## KEY POINTS

Remote units were established in two Norwegian districts

- The units included a nurse and video link to the doctor on call at a casualty clinic
- Contact and consultation rates decreased in the municipalities with remote units
- Use of the remote units was low in both participating areas
- Home visits and callouts were substantially reduced in the district where remote units replaced a doctor on-call

## Introduction

Norway has a spread settlement with large rural areas in most of the country. The mean number of inhabitants in a Norwegian municipality is 15,000 and the median is only 5,100. During the last decade several municipalities have merged, reorganised their services or created inter-municipal cooperations to run their services. So also with primary emergency care out-of-hours (OOH). The number of OOH districts in Norway has decreased from 230 in 2007 to 168 in 2022 [1]. With larger OOH districts the consequences are often the same; the OOH district with a belonging casualty clinic serves a larger geographical area and population than before. This implies longer distances for patients in the outskirts to reach the casualty clinic and home visits and response time in emergencies for doctors on call become longer.

These developments have challenged the emergency medical system (longer response times) and patients’ satisfaction with the services (longer travel times for consultations). It has been recommended that 90% of the population within an OOH district should reach their casualty clinic within 40 min of travel time [2], but this aim is not fulfilled in many areas. Doctors, municipalities, and agencies have discussed possible compensatory actions to increase the availability of medical assessments within a reasonable time and increase the quality and availability of prehospital emergency services. Direct attendance at the casualty clinics is neither common nor recommended in Norway. Inhabitants are encouraged to call the local emergency communication centre (LEMC) by the national OOH number 116117, and a nurse then decides the needed level of care by telephone triage. Emergency departments at hospitals are not open for direct attendance.

The use of video in rural areas has been considered useful for healthcare providers and patients [3]. It is particularly beneficial for the triage of urgent patients to select the right level of care [4]. Video consultations may substitute patient transport to a hospital or casualty clinic [5] and provide good medical support to local healthcare providers in diagnostics and monitoring [5–7].

In 2018, the Norwegian Directorate of Health proposed a new OOH concept consisting of OOH units for video consultations in remote areas in two large OOH districts, thus aiming at increasing the availability of services and reducing transportation time for patients (personal communication). A research project was launched to investigate the new model, including the use of the new service, the kind of medical problems handled, and if this kind of service changed rates of contacts, home visits, and consultations.

This article reports observational data from the two OOH districts, based on registrations before and after introducing the new remote OOH units.

## Materials and methods

### Study setting

The Norwegian Directorate of Health selected two OOH districts in Western Norway to test the concept with remote OOH units (Table 1), after a self-nomination process. The Directorate funded the project, including the evaluation project. The National Centre for Emergency Primary Health Care was given responsibility for planning the data collection and subsequent analyses of data (first author EZ as project leader), while the two OOH districts were responsible for establishing the remote OOH units, including organization, management, employment, and running the units at a daily basis.

**Table 1.** Populations and travel times by car for the two OOH districts with remote OOH units to the casualty clinic in Førde and Molde.

District	Remote units and controls		Population	Travel time by car (minutes)
**Førde**			38 000	–
	Remote unit I	Balestrand	1 300	70–104
	Remote unit II	Høyanger	4 100	55–72
	Remote unit III	Hyllestad	1 300	77–83
	Remote unit IV	Askvoll	3 000	45–62
	Remote unit V	Bremanger**	3 700	75–130
	Control areas	Førde area	24 600	0–90
**Molde**			63 300	–
	Remote unit I	Midsund*	2 000	33–59
	Remote unit II	Aukra*	3 600	15–60
	Remote unit III	Sunndal	7 100	70–115
	Remote unit IV	Rauma*	7 500	57–129
	Control	Molde area	43 100	0–61

Data from 2021.

*A ferry transport is necessary. **Not part of the inter-municipal cooperation, as the rest is.

Førde OOH district covers nine municipalities with a geographical area of 5 457 km^2^ around the city of Førde, with a single casualty clinic in Førde connected to the local hospital. This OOH district was established in 2009, and the inhabitants had to drive to Førde, often more than one hour travel time. The establishment of remote OOH units could thus be seen as a new local OOH service with higher accessibility and shorter transport and response time. Remote OOH units were established in four municipalities while the five other municipalities were defined as control municipalities. Additionally, one municipality outside Førde OOH district replaced their casualty clinic with a remote OOH unit at nighttime. There was a total of 38,000 inhabitants, while municipalities with remote OOH units included 13,400 inhabitants (Table 1). At the well-equipped casualty clinic in Førde, including a rapid response car, there were two primary care doctors on call 24/7, mostly local GPs. The clinic is co-located with the local emergency communication centre (LEMC), staffed with registered nurses.

Molde OOH district covers eight municipalities with a geographical area of 5,250 km^2^ around Molde city, including several settlements on islands, with a single casualty clinic in Molde. Four municipalities established remote OOH units, and four were defined as control municipalities (Table 1). The Molde OOH district had 63,300 inhabitants, while the municipalities with remote OOH units had 20,200. In Molde OOH district, in contrast to Førde OOH district, four local casualty clinics were shut down and replaced with remote OOH units in the municipalities, by establishing a new and large OOH district with a central casualty clinic in Molde. However, a GP was still present for two hours in the afternoon during weekdays and three hours during Saturdays, Sundays, and holidays. The new organization with remote OOH units could thus be seen as a reduction in the emergency care services and OOH availability in these municipalities. At the well-equipped casualty clinic in Molde, including a rapid response car, there were two doctors on call 24/7, mostly local GPs. The local emergency communication centre (LEMC) is located at the hospital in Molde, staffed with registered nurses.

### Description of remote OOH units

Each remote OOH unit was organized as part of the OOH districts in Førde and Molde. Most often the remote OOH units were a room in the local nursing home. It was equipped with a video link system, some basic diagnostic equipment for clinical examinations, and a variety of basic laboratory and technical equipment, for example, ECG, blood pressure device, oxygen saturation (SpO_2_,), urine dipstick, u-HCG, micro-swabs, rapid Strep A test, CRP, Hb, blood sugar, and venipuncture. Drugs commonly used at OOH clinics were also available [8].

A nurse was available for the patients at each remote OOH unit, from 4 pm until 8 am (10 pm to 8 am in one municipality), on weekdays and 24 h on Saturday, Sunday, and other holidays. The nurses were usually working normal shifts at the local nursing home. The nurse would open the remote OOH unit for a new patient after notification from the LEMC. In the remote OOH units in the Førde OOH district, the nurses were extra in the rotation system and were free to leave when a patient should meet at the remote OOH unit. In the Molde OOH district, the nurses were part of the normal rotation system and had to leave ordinary work to open the remote OOH units. The nurses got extensive repetition and training in emergency medicine to achieve the required skills [9].

The inhabitants of the municipalities with remote OOH units were informed about the service through the municipalities’ websites, local newspapers, and other channels, but should otherwise make contact with the LEMC. The triage nurse at the LEMC evaluated the call and the medical problem and decided if the case was suitable for a consultation at the remote OOH unit (Figure 1). If a remote consultation was offered and accepted, the patient could visit the remote OOH unit. Then, a video consultation with the GP on call at the casualty clinic was set up, and the present local nurse could do physical examinations, laboratory tests, or therapeutic interventions on the patient, based on what the doctor found necessary and relevant.

Based on the history and clinical or laboratory findings the doctor then decided further management of the patient; including referral to the hospital, further investigations after transport to the casualty clinic, or returning home with or without medication or other treatment, and/or further follow up by local GP the next day(s).

### Outcomes and data collection

A prospective observational study was implemented to evaluate the number of contacts to the LEMCs in the two OOH districts. We analysed the distribution of different types of consultations with the on-call GP, by data from municipalities with a remote OOH unit and a group of control municipalities. ICPC-2 codes defined medical problems. All contacts to the two LEMCs in Førde and Molde were registered between 3 pm and 8 am on weekdays and 24 h at weekends and public holidays. The Control and Payment of Reimbursement to Health Service Providers database (KUHR) gave data on all patient contacts with the GPs on call. Based on payment codes, data on face-to-face or video consultations, home visits and callouts in municipalities with remote OOH units and control municipalities could be collected.

**Figure 2.**
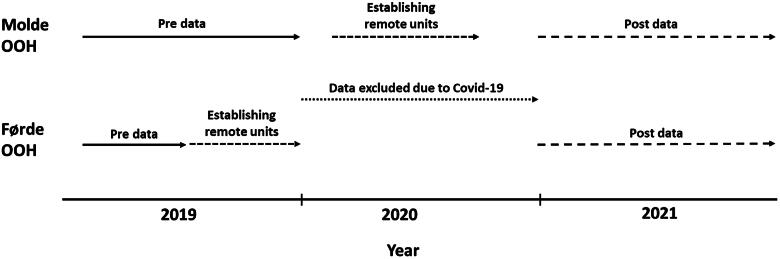
Timelines for data collection and establishing remote OOH units in molde and førde districts.

The data presented from Førde LEMC and KUHR are from seven months before the remote OOH units opened (1^st^ of February 2019 until 31^st^ of August 2019), and 12 months in 2021. The OOH units were established during autumn 2019, the first on the 1^st^ of September. The data presented from Molde LEMC and KUHR are from eleven months before the units opened (1^st^ of February 2019 until 31^st^ of December 2019), and 12 months in 2021. Two of the remote OOH units were established in February 2020, and two in September 2020. Figure 2 shows the timeline for data collection and establishing the remote OOH units.

**Figure 1.**
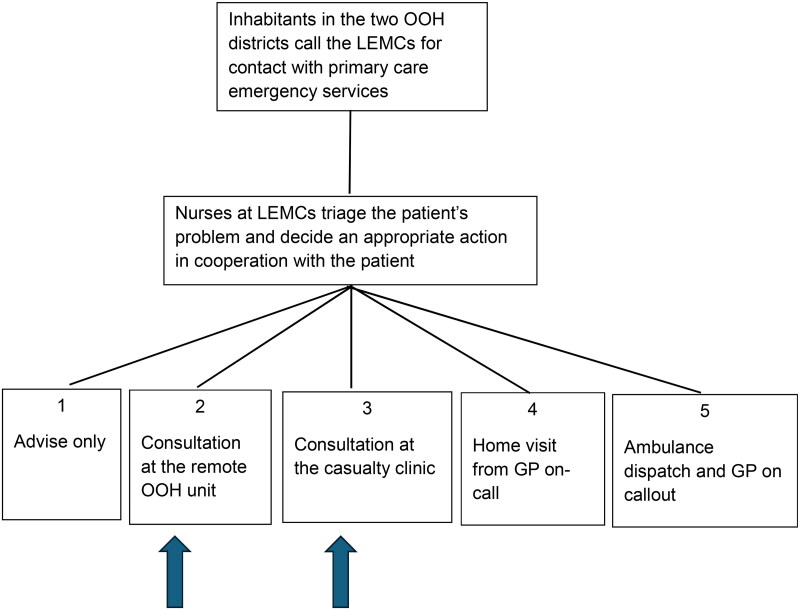
Possible first actions for inhabitants after contacting the LEMCs by telephone number 116 117. The two arrows show where there was an expected change in the distribution of consultations as a result of the implementation of remote units. There are further options after the first action: a consultation at a remote OOH unit may result in hospitalization. Consultations at a casualty clinic often end with the patient travelling home [1,9,20].

Data from 2020 are excluded from both areas due to the COVID-19 outbreak and its concomitant strong confounding on contacts to the LEMCs [10,11]. All the remote OOH units were closed for patients with airway symptoms in 2020. The municipality of Midsund was merged with Molde in January 2020. This is adjusted for when rates and 95% confidence intervals are calculated.

### Statistics

Statistical analyses were performed using IBM SPSS Statistics 27.0. Data on OOH contacts and payment codes are presented as figures and rates per 1,000 inhabitants per year, with a 95% confidence interval (CI). To test differences in contacts and types of consultations between 2019 and 2021, a chi-squared tests were used. Differences are regarded as statistically significant if *p* < 0.05. In Førde data from the seven months pre-project period were transformed to annual rates by multiplying by 12/7. Similarly, the pre-project period in Molde was 11 months, and annual rates were calculated by multiplying by 12/11.

## Results

Table 2 shows the calculated numbers and rates of contacts to the LEMCs in Førde and Molde during the baseline periods in 2019 compared with 2021; in total, by municipalities with remote OOH units combined, and for control municipalities. Førde LEMC had an 11% higher total pre-project rate than Molde LEMC. In 2021, the total contact rates had decreased by about 3% in Molde (*p* < 0.05) and <1% in Førde (*p* > 0.05), respectively. In 2019, there was a statistically significant higher contact rate from municipalities where remote OOH units should be established, in Førde OOH district (+3%) compared with control municipalities. In Molde OOH district, there were no statistically significant differences (+2%) between municipalities where remote OOH units should be established and control municipalities.

**Table 2.** The total number of contacts to the emergency number 116 117 to the LEMCs in Førde and Molde, by municipalities with remote units and control municipalities.

	Year 2019 (baseline)*			Year 2021		
Period	N	Rate	95 % CI	N	Rate	95 % CI
**Førde**						
Total	14 348	377	371–384	14 207	374	368–380
Remote units	5 167	386	375–396	4 416	330	320–339
Controls	9 182	373	366–381	9 791	398	390–406
**Molde**						
Total	21 314	337	332–341	20 647	326	322–331
Remote units	6 860	340	332–348	5 230	287	280–295
Controls	14 454	335	330–341	15 417	358	(352–363)

The rate is calculated as contacts per 1,000 inhabitants per year in 2019 and 2021, with a 95% CI.

*Calculated based on figures for contacts in the period from 1. February until 31. August 2019, in the Førde area. Calculated based on figures for contacts in the period from 1. February until 31. December 2019, in the Molde area.

From 2019 to 2021, Førde LEMC had a statistically significant decrease of 15% in contact rates from inhabitants in municipalities with remote OOH units and a statistically significant increase of 6% in the control municipalities (Table 2). In Molde LEMC, there was a similarly statistically significant decrease of 16% in contact rates from inhabitants in municipalities with remote OOH units. In comparison, there was a small, but statistically significant increase of 2% in contacts from the control municipalities (Table 2). This resulted in a 17% and 16% difference in 2021 rates between control municipalities and municipalities with remote OOH units in Førde and Molde OOH districts, respectively.

In 2019, 51% of all contacts (Table 2) to the LEMCs from municipalities that were going to establish a remote OOH unit, resulted in a consultation with the GP on-call, including home visits and callouts (Tables 2 and 3). In 2021, when the remote OOH units had been established, the percentage was the same in the Førde OOH district. However, the rate of consultations decreased by 16% from 2019 to 2021 (Table 3). In the Molde OOH district, there were no differences in the percentage of consultations after contact, between 2019 and 2021 in municipalities with remote OOH units (Tables 2 and 3). However, the total consultation rate decreased by 12% from 2019 to 2021 (Table 3). For the control municipalities in the Førde OOH district, contacts that ended with a consultation were 53% in 2019 and 45% in 2021 (Tables 2 and 3). This corresponds to an 8% reduction in the consultation rate (*p* < 0.05) (Table 3). In the Molde OOH district, the figures from 2019 were 62% and in 2021, 57%, corresponding to a 6% reduction in consultation rate (*p* < 0.05).

**Table 3.** Consultations, home visits, and emergency callouts in remote units and controls in Førde and Molde.

	2019			2021		
Period	N	Rate	95 % CI	N	Rate	95 % CI
**Førde**						
Total	7 516	198	193–202	6 672	176	171–180
Video consultations at remote unit areas	0			300	22	20–25
Ordinary consultations at OOH clinic*	2 478	185	178–192	1 798	134	128–140
Ordinary consultations control areas	4 675	190	185–195	4 300	175	170–180
Home visits in remote unit areas**	169	13	11–15	127	11	9–12
Home visits in control areas	146	6	5–7	128	5	4–6
Emergency call-outs in remote unit areas**	11	1	0–1	9	1	0–1
Emergency call-outs in control areas	10	1	0–1	10	0	0–1
**Molde**						
Total	12 638	200	196–203	11 715	185	182–188
Video consultations at remote unit areas	0			161	8	7–9
Ordinary consultations at OOH clinic*	3 183	155	150–161	2 604	143	138–149
Ordinary consultations control areas	8 758	203	199–207	8 670	192	188–196
Home visits in remote unit areas**	445	22	20–24	109	6	5–7
Home visits in control areas	183	4	4–5	161	4	3–4
Emergency call-outs in remote unit areas**	44	2	2–3	6	0	0–1
Emergency call-outs in control areas	25	1	0–1	4	0	0

Midsund was included as a control municipality for Molde in 2021. The rate is calculated as events per 1,000 inhabitants per year.

*Ordinary consultations among inhabitants from the municipalities with a remote unit, who have met at the OOH clinic in Førde or Molde.

**The municipality of Bremanger in the Førde area, represented 53% of all home visits and 91% of all call-outs in remote unit areas in 2019. In 2021, Bremanger represented 100 % of all home visits and call-outs.

In 2021, 7% of all contacts to Førde LEMC from inhabitants in municipalities with remote OOH units, were handled by the remote OOH units. In the Molde OOH district, the percentage was 3%. When a consultation was needed, including home visits, 13% were handled at remote OOH units in the Førde OOH district, in the Molde OOH district the share was 6%.

In the Molde OOH district where municipalities with ordinary casualty clinics staffed with a doctor on call were replaced with remote OOH units, the number of home visits showed a statistically significant reduction of 76% from 2019 to 2021. Similarly, callouts had a statistically significant reduction of 86%. There were no changes in the Førde OOH district (Table 3).

Table 4 shows ICPC-2 diagnosis chapters used after consultations in the municipalities with remote OOH units in 2021. The most striking differences in the chapter distributions are a much higher use of Chapter A/General and unspecified) in Molde OOH district and a much higher use of Chapter R (Respiratory) in Førde OOH district.

**Table 4.** Diagnoses by ICPC-2 chapters after consultations in remote units (video consultations) in 2021. Presented as numbers and per cent.

	Molde		Førde	
	N	Per cent	N	Per cent
**ICPC – Chapters N (%)**				
A: General and unspecified	38	24	34	11
D: Digestive	14	9	19	6
K: Cardiovascular	4	2	7	3
L: Musculoskeletal	17	11	16	5
P: Psychological	17	11	8	3
R: Respiratory	34	21	122	41
S: Skin	10	6	18	6
U: Urological	12	7	16	5
Other chapters	15	9	60	20
** SUM**	**161**	**100**	**300**	**100**

## Discussion

Both Førde and Molde OOH districts were able to establish the planned remote units. They could all offer the intended services to the local populations, for example, video consultations, nurse-led clinical investigations, and laboratory tests, and when necessary, also home visits and emergency callouts by the doctor on call at the casualty clinic. Changes were found in the number of contacts and the use of services in the new remote OOH units compared with the pre-project period, of which some were similar for the two districts while some changes were very different in Førde and Molde OOH districts. Contacts to the LEMCs and doctor consultations decreased in the municipalities with remote OOH units. Only a small share of the contacts to the LEMCs resulted in activities at the remote OOH unit level; in Førde 7% and in Molde only 3%. Home visits and callouts decreased dramatically in the Molde district after that casualty clinics were replaced with remote OOH units, indicating a decreased commitment to emergency medicine regulatory requirements.

### Strengths and weaknesses of the study

A strength of this study is that we have a complete registry data set for reimbursement codes for consultations during the study period and thus could compare the same variable in a before-after design. However, we had to calculate the number of contacts and consultation types to obtain complete 2019 data, which may somewhat affect the accuracy of the data. This applies in particular to Førde district, where ‘before data’ period was 7 months. Season variations like infections during autumn and the first winter months may be lost. Still, the data from February until April will include winter conditions. The number of contacts is based on manually extracted data from the LEMCs system, and some missing data may have reduced the number of included calls. However, such potential reduction registrations should similarly affect municipalities with or without remote OOH units. We have registered visits and consultations taking place, we cannot report visits offered but not accepted at the remote OOH clinics. Likewise, patients offered but declined a consultation with a GP at the casualty clinic were not registered. The Covid-19 pandemic had an impact on inhabitants’ healthcare seeking also in 2021, but there is no reason to believe that this had a different impact within different municipalities. However, due to the unknown but possible effect of Covid-19, it is difficult to establish causality between establishing remote OOH units and the reduction of contacts and different consultation types between 2019 and 2021.

#### Interpretation of findings

The total number of contacts to the LEMCs showed a minor decrease in 2021 compared with 2019, while the number of contacts from municipalities with remote OOH units showed a reduction. National data from Norway shows a six percent reduction in OOH consultation rates from 2019 to 2021 [12], for all types of consultations combined. This decline is in line with the decline of consultations in the control municipalities. It is known that the COVID-19 outbreak led to reduced contacts with infectious diseases to LEMCs and casualty clinics, and this effect might partly be present also in 2021 [13].

Studies from Norway and England showed that the number of contacts to the OOH service decreased with a longer distance to the casualty clinics [14,15]. Remote OOH units manned with nurses were meant to be a nearby and better service, thus perhaps being an incitement for an increased number of contacts. This was not experienced in the Førde OOH district. In the Molde OOH district, however, where remote OOH units replaced doctor-staffed clinics in the municipalities, the consequence was a much longer distance for many inhabitants to the casualty clinic in Molde. This may explain the reduction of contacts to the LEMC from the municipalities with remote OOH units. Overall, our results indicate that establishing remote OOH units could have a negative impact on contact rates in both areas, although not very large.

The idea of establishing remote OOH units follows a long-lasting pressure to reduce the workload for GPs [16,17]. Thus, the doctors can be on call more seldom at a central-based casualty clinic, while patient services can be partly upheld by video consultations and locally administered nurse-led investigations and tests. An important disadvantage with larger geographical areas is long driving time for the patients, with a danger of less use of the casualty clinic, and also for serious symptoms [14]. While the aim of a reduced workload was fulfilled, only a small fraction of the patients were handled by the remote OOH units, neither by video nor by local attendance. In 2021, most patients who needed a consultation still met at the casualty clinics, not at the remote OOH units. If the establishment of the remote OOH units is the cause, it should lead to a fundamental discussion about the balance between advantages and costs for the investigated model. In addition to attendance at the casualty clinics, phone calls may be concluded by nurses’ advice and no further actions taken. This is important to reduce unnecessary patient attendance (Tables 2 and 3). Nurse-led telephone triage and counselling are normal practices also in other countries [18,19].

Another probable side effect was the impact on home visits and callouts. A major prerequisite aimed by The Norwegian Directorate of Health, was that the emergency services should be upheld or preferably strengthened [3]. The municipalities with remote OOH units in Molde had high rates of home visits and callouts pre-project, thus complying with recommendations [2]. In Førde OOH district, established in 2009, 2019 rates were already low, and most activity was in one municipality, not part of the Førde OOH district. Establishing remote OOH units seems not to affect the low rates of home visits and callouts in Førde. In the Molde OOH district, however, establishing remote OOH units instead of local casualty clinics, would be the most probable explanation for the negative impact on rates of home visits and callouts. From GPs doing home visits and callouts within the recommended range per 1,000 inhabitants in 2019 [2], the new organization could have led to a large reduction of emergency services in municipalities with remote OOH units. In 2021, patient events with a high degree of urgency in the municipalities with remote OOH units were left to the ambulance service alone, without the support of a present GP. Thus, the responsible municipalities do not take part in their emergency medical responsibility together with the specialist health service (ambulances and hospital trusts). Our results indicate that the decentralized remote OOH units did not compensate for the centralization of casualty clinics, neither by offering video consultations nor by home visits or callouts. Irrespective of how the remote OOH units influenced consultation types, it must be seriously considered if the remote municipalities in both Førde and Molde comply with National emergency medical regulatory requirements.

ICPC diagnoses were recorded for all types of consultations in municipalities with remote OOH units. The two districts differed from each other and were also compared with national data [20]. In the Molde area use of Chapter A was 37% higher and Chapter P 45% higher, compared with national data, while the use of Chapter K was 31% lower. In the Førde area use of Chapter R was 49% higher, compared with national data. The differences indicate that remote OOH units were used differently in the two districts, possibly influenced by local traditions and policies, thus making analyses and generalization difficult.

## Conclusion

It is not possible to establish a certain causality between the remote OOH units and the differences between 2019 and 2021 data. The rate of contacts from inhabitants in municipalities decreased in 2021. The vast majority of all contacts to the LEMCs were handled with other actions than a video consultation at the remote OOH units. In the Molde OOH district there was a large reduction of home visits and callouts in 2021, compared with 2019. One possible explanation could be the replacement of local doctor-staffed casualty clinics with remote OOH units.

## References

[1] Allertsen M, Morken T. Legevaktorganisering i Norge. Rapport fra Nasjonalt legevaktregister 2022. Rapport nr. 4-2022. Bergen Nasjonalt kompetansesenter for legevaktmedisin, NORCE Norwegian Research Centre; 2022. [Emergency Primary Care organization in Norway]. Norwegian. Available from: http://Legevaktorgan­seringiNorge.RapportfraNasjonaltlegevaktregister2022.pdf (norceresearch.no) [cited 2024 Sept 16].

[2] Ministry of Health and Care Services. NOU. 2015:17. Først og fremst. [Norwegian public report ; 2015:17. A comprehensive system for handling acute diseases and out-of-hospital injuries]. Norwegian. Available from: NOU 2015: 17 (regjeringen.no) [cited 2024 Sept 16].

[3] Johansson AM, Lindberg I, Söderberg S. Healthcare personnel’s experiences using video consultation in primary healthcare in rural areas. Prim Health Care Res Dev. 2017;18(1):73–83. doi: 10.1017/S1463423616000347. 27640522

[4] Kim Y, Groombridge C, Romero L, et al. Decision support capabilities of telemedicine in emergency prehospital care: systematic review. J Med Internet Res. 2020;22(12):e18959. doi: 10.2196/18959. 33289672 PMC7755537

[5] O’Sullivan SF, Schneider H. Developing telemedicine in emergency medical services: a low-cost solution and practical approach connecting interfaces in emergency medicine. J Med. 2022;10:6. 10.1177/27550834221084656 PMC9413501 36204523

[6] Vicente V, Johansson A, Selling M, et al. Experience of using video support by prehospital emergency care physician in ambulance care - an interview study with prehospital emergency nurses in Sweden. BMC Emerg Med. 2021;21(1):44. doi: 10.1186/s12873-021-00435-1. 33827436 PMC8028766

[7] Kirk UB, Payne R, Tweedie J, et al. ‘A tool for every job’: use of video in urgent primary care. BJGP Open; 2024;74:443–444. doi: 10.3399/bjgp24X739473.[cited 2024 Des 30]. PMC11441613 39327075

[8] Rebnord IK, Thue G, Hunskår S. Equipment, laboratory analyses and drugs in out-of-hours services in Norwegian municipalities. Tidsskr nor Legeforen. 2009;129:987–990. 10.4045/tidsskr.08.0288 19448751

[9] Ministry of Health and Care Services. Forskrift om krav til og organisering av kommunal legevaktordning, ambulansetjeneste, medisinsk nødmeldetjeneste mv. [Regulations on requirements for and organization of out-of-hour services, ambulance services, emergency medical services etc; 2015 ]. Norwegian. Available from: https://lovdata.no/dokument/SF/forskrift/2015-03-20-231?q=akuttmedisinforskriften. [cited 2024 Sept 16].

[10] Midtbø V, Johansen IJ, Hunskaar S. The association between municipal pandemic response and COVID-19 contacts to emergency primary health care services: an observational study. BMC Health Serv Res. 2023;23(1):479. doi: 10.1186/s12913-023-09489-2. 37170224 PMC10175054

[11] Huibers L, Bech BH, Kirk UB, et al. Contacts in general practice during the COVID-19 pandemic: a register-based study. Br J Gen Pract. 2022;72(724):e799–e808. [cited 2024 Sept 16]. doi: 10.3399/BJGP.2021.0703. 36253113 PMC9591020

[12] Statistics Norway. ; 2014. GPs and emergency primary health care. Available from: 10903: emergency Primary Health Care consultations, by age, sex and diagnosis - 2023. Statbank Norway (ssb.no) [cited 2024 Sept 16].

[13] Norwegian Institute of Public Health. 2021 Helse og omsorgstjenester under pandemien. [Health and care services during the Covid pandemic]. Norwegian. Available from: del 8: helse- og omsorgstjenestene under pandemien - FHI [cited 2024 Sept 16].

[14] Raknes G, Holm Hansen E, Hunskaar S. Distance and utilisation of out-of-hours services in a Norwegian urban/rural district: an ecological study. BMC Health Serv Res. 2013;13(1):222. doi: 10.1186/1472-6963-13-222. 23773207 PMC3703450

[15] Turnbull J, Martin D, Lattimer V, et al. Does distance matter険 Geographical variation in GP out-of-hours service use: an observational study. Br J Gen Pract. 2008;58(552):471–477. doi: 10.3399/bjgp08X319431. 18611312 PMC2441507

[16] Morken T, Rebnord IK, Maartmann-Moe K, et al. Workload in Norwegian general practice 2018 – an observational study. BMC Health Serv Res. 2019;19(1):434. doi: 10.1186/s12913-019-4283-y. 31253160 PMC6599272

[17] Rebnord IK, Morken T, Maartmann-Moe K, et al. Out-of-hours workload among Norwegian general practitioners – an observational study. BMC Health Serv Res. 2020;20(1):944. doi: 10.1186/s12913-020-05773-7. 33054822 PMC7557051

[18] van Charante EPM, ter Riet G, Drost S, et al. Nurse telephone triage in out-of-hours GP practice: determinants of independent advice and return consultation. BMC Fam Pract. 2006;7(1):74. doi: 10.1186/1471-2296-7-74. 17163984 PMC1713241

[19] Purc-Stephenson RJ, Thrasher C. Patient compliance with telephone triage recommendations: a meta-analytic review. Patient Educ Couns. 2012;87(2):135–142. doi: 10.1016/j.pec.2011.08.019. 22001679

[20] Sandvik H, Hunskår S, Blinkenberg J. ; 2022Årsstatistikk for legevakt 2021. [Statistics from out-of-hours primary health care 2021]. Report no. 1-2022. Bergen: National Centre for Emergency Primary Health Care, NORCE Norwegian Research Centre,. Norwegian. Available from: https://norceresearch.brage.unit.no/norceresearch-xmlui/bitstream/handle/11250/2989361/%25C3%2585rsstatistikk_fra_legevakt_2021.pdf?sequence=1. [cited 2024 Sept 16].

